# Seasonal regulation of singing-driven gene expression associated with song plasticity in the canary, an open-ended vocal learner

**DOI:** 10.1186/s13041-021-00869-5

**Published:** 2021-10-29

**Authors:** Shin Hayase, Chengru Shao, Masahiko Kobayashi, Chihiro Mori, Wan-chun Liu, Kazuhiro Wada

**Affiliations:** 1 Graduate School of Life Science, Hokkaido University, Sapporo, Hokkaido Japan; 2 Department of Psychology, Colgate University, Hamilton, NY USA; 3 Department of Biological Sciences, Hokkaido University, Sapporo, Hokkaido Japan; 4 Faculty of Science, Hokkaido University, North 10, West 8, Kita-ku, Sapporo, Hokkaido Japan; 5 Present Address: Department of Molecular Biology, Faculty of Pharmaceutical Sciences, Teikyo University, Kaga, Itabashi-ku, Tokyo, Japan

**Keywords:** Song learning, Sensorimotor learning, Learning plasticity, Critical period, Arc, IEGs

## Abstract

Songbirds are one of the few animal taxa that possess vocal learning abilities. Different species of songbirds exhibit species-specific learning programs during song acquisition. Songbirds with open-ended vocal learning capacity, such as the canary, modify their songs during adulthood. Nevertheless, the neural molecular mechanisms underlying open-ended vocal learning are not fully understood. We investigated the singing-driven expression of neural activity-dependent genes (*Arc*, *Egr1*, *c-fos*, *Nr4a1*, *Sik1*, *Dusp6*, and *Gadd45β*) in the canary to examine a potential relationship between the gene expression level and the degree of seasonal vocal plasticity at different ages. The expression of these genes was differently regulated throughout the critical period of vocal learning in the zebra finch, a closed-ended song learner. In the canary, the neural activity-dependent genes were induced by singing in the song nuclei throughout the year. However, in the vocal motor nucleus, the robust nucleus of the arcopallium (RA), all genes were regulated with a higher induction rate by singing in the fall than in the spring. The singing-driven expression of these genes showed a similar induction rate in the fall between the first year juvenile and the second year adult canaries, suggesting a seasonal, not age-dependent, regulation of the neural activity-dependent genes. By measuring seasonal vocal plasticity and singing-driven gene expression, we found that in RA, the induction intensity of the neural activity-dependent genes was correlated with the state of vocal plasticity. These results demonstrate a correlation between vocal plasticity and the singing-driven expression of neural activity-dependent genes in RA through song development, regardless of whether a songbird species possesses an open- or closed-ended vocal learning capacity.

**Supplementary Information:**

The online version contains supplementary material available at 10.1186/s13041-021-00869-5.

## Introduction

Learned behaviors have species-specific features that have originated owing to species differences in the structure and physiological function of neural circuits for generating associated behavior [1–5]. However, the detailed neural molecular mechanisms underlying species-specific learned behaviors have not been fully clarified. To tackle this issue, oscine songbirds have been used as a salient model system owing to their unique song-learning ability, which is species-specifically regulated through conserved neural circuits, called song circuits, for song learning and production [1, 6–9].

**Fig. 1:**
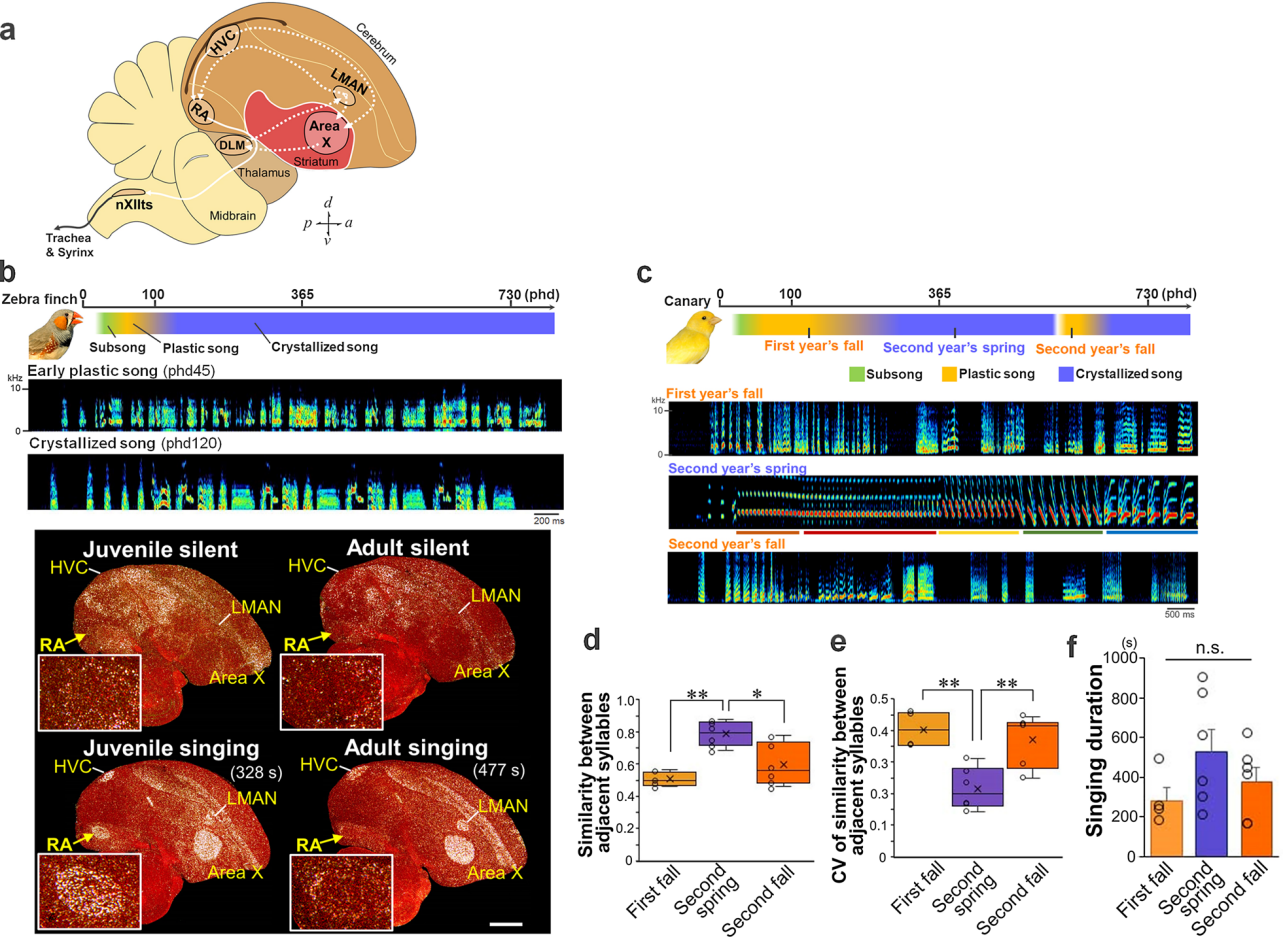
Seasonal song plasticity in the canary. **a** Diagram of the song circuits. The posterior vocal pathway and the anterior forebrain pathway (pallial–basal ganglia–thalamic circuit) are represented as solid and dotted white lines, respectively. HVC (proper name); RA, robust nucleus of the arcopallium; Area X, Area X of the striatum; DLM, dorsal lateral nucleus of the medial thalamus; LMAN, lateral magnocellular nucleus of the anterior nidopallium; nXIIts, tracheosyringeal portion of the hypoglossal nucleus. d/v/a/p, dorsal/ventral/anterior/posterior. **b** (Top) Timeline of song sensorimotor learning period of the zebra finch, a closed-ended vocal learner. phd, post-hatching day. (Middle) Early plastic and crystallized songs of a zebra finch. (Bottom) Typical singing-driven expressions of *Arc* mRNA (white) in the song nuclei, HVC, RA, LMAN, and Area X of zebra finch juveniles (silent, 52 phd; singing, 47 phd) and adults (silent, 124 phd; singing, 112 phd). Total singing duration(s) before brain sampling is shown in the panel. White dots: *Arc* mRNA expression. Red: cresyl violet counterstain. Scale bar = 1.5 mm. *Arc* expressions in RA were enlarged with a white outline. All images are adapted from Hayase et al. 2018 PLoS Biology [41]. **c** (Top) Timeline of the song sensorimotor learning period of the canary, an open-ended vocal learner. (Bottom) Seasonal changes in song plasticity of canary songs. Colored lines: crystallized repetitive phrases of the structured song in second spring. **d** Mean similarity scores between adjacent syllables in first fall (n = 4 birds), second spring (n = 6 birds), and second fall (n = 6 birds) (300 syllable sets/bird). **e** CV of similarity scores between adjacent syllables in first fall, second spring, and second fall. For (D) and (E), one-way ANOVA with Scheffe’s F test. *p < 0.05, **p < 0.01. **f** The mean cumulative singing duration (sec) in 30-min singing sessions before brain sampling from singers in first fall (n = 4 birds), second spring (n = 6 birds), and second fall (n = 6 birds). One-way ANOVA. ^n.s.^p > 0.05

The song circuits in songbirds comprise discrete, well-defined forebrain regions known as song nuclei that have comparable characteristics in terms of topological and anatomical connectivities across species (Fig. 1a). These song nuclei are subdivided into two main pathways: the vocal motor pathway (VMP) and the anterior forebrain pathway (AFP). The VMP, which is similar to mammalian motor pathways, comprises the vocal premotor nuclei HVC (used as a proper name) and the robust nucleus of the arcopallium (RA) [10]. RA projects to the tracheosyringeal portion of the hypoglossal nucleus (nXIIts) that connects to syringeal muscles [11]. RA and HVC contribute to the regulation of the acoustic features (syllables) and the temporal pattern (sequence) of the song, respectively [12–14]. The AFP, which forms a pallial (cortical)–basal ganglia–thalamic loop, is a key site for generating vocal exploratory fluctuations for song learning [15–18]. It comprises the lateral magnocellular nucleus of the anterior nidopallium (LMAN), basal ganglia nucleus Area X, and the dorsal lateral nucleus of the medial thalamus (DLM). The output activity of the AFP is conveyed from LMAN to RA. Thus, the motor nucleus RA is an essential site for integrating two neural transmissions—one from the upstream vocal motor nucleus HVC and the other from the AFP output nucleus LMAN.

The timing and degree of vocal plasticity for song acquisition are prominent species-specific features that are differently regulated throughout the lifespans among songbird species. Closed-ended (also called age-limited) song learners, such as zebra finches (*Taeniopygia guttata*) and Bengalese finches (*Lonchura striata* var. *domestica*), have a single sensitive period for song learning after hatching, i.e., they do not change their song after its crystallization at the adult stage (Fig. 1b) [19, 20]. By contrast, open-ended song learners, such as canaries (*Serinus canaria*) and European starlings (*Sturnus vulgaris*), can modify their songs during adulthood [21–26]. In the canary, during the first year after hatching, juveniles begin singing subsongs in the summer that gradually develop into louder, more structured songs (termed plastic songs) in the fall. The plastic song becomes crystallized in a process lasting until late winter, and the birds continue to sing the crystallized songs until the following spring and early summer as adults (Fig. 1c). Crystallized songs of the canary are structured with multiple phrases that form clusters of repetitive syllables. By the fall of the second year, their songs gradually deteriorate to the plastic song structure again, after which they recrystallize their songs with some modification by the late winter of the second year (Fig. 1c). This annual regulation, involving cycles of song degradation and crystallization, continues after the second year. In the canary brain, a greater number of new neurons are generated and incorporated into HVC in the fall than in the spring [27, 28]. Such newly added neurons are replaced as RA-projecting excitatory neurons in HVC (HVC_(RA)_ neurons) [29, 30], leading to the hypothesis that seasonal regulation of neurogenesis and subsequent replacement with new HVC_(RA)_ neurons could be important factors for open-ended vocal learning [28, 31, 32]. However, the latent contribution of other brain sites and its relationship with neurogenesis in HVC are not fully examined.

Neuronal activity induces a genetic response, called activity-dependent transcription, in the brain. The neural activity-dependent genes, e.g., immediate-early genes (IEGs), either directly or indirectly influence the physiological function and structural maturation of neural circuits as genetic regulators for long-term neuronal plasticity [33–35]. Singing behavior also induces a set of activity-dependent genes in the song nuclei of songbirds [36–43]. In zebra finches, subsets of singing-driven neural activity-dependent genes are highly expressed in the projection neurons of the vocal motor nucleus RA in juveniles with high vocal plasticity compared with adults with crystallized songs (Fig. 1b) [41, 44, 45]. Despite singing prevention during the critical period of vocal motor learning, juvenile-like intense expression of the singing-driven genes persists in adults. Additionally, birds produce a juvenile-like plastic song and retain sufficient ability to acquire a tutored song even at adulthood when allowed to sing freely despite singing prevention during the critical period [41]. On the basis of these findings, we hypothesized that the expression level of singing-driven neural activity-dependent genes contributes to the degree of song plasticity in open-ended song learners in an age-independent manner. In this study, we evaluated this possibility by assessing whether adult canaries possess the ability to induce a high expression level of neural activity-dependent genes, as do juveniles. For this purpose, the expression of neural activity-dependent genes (*Arc*, *Egr1*, *c-fos*, *Nr4a1*, *Sik1*, *Dusp6*, and *Gadd45β*) was examined in the brains of canaries. These genes were identified to be differently induced by singing in the RA of juvenile and adult zebra finches (Fig. 1b: *Arc* as an example) [41]. We collected brain samples at two seasonal time points from canaries with different ages and vocal plasticity states: (i) juveniles with plastic songs in the fall of the first year (first fall), (ii) adults with crystallized songs in the spring of the second year (second spring), and (iii) adults with plastic songs in the fall of the second year (second fall). Furthermore, to exclude the potential effects of neuronal cell density on signal detection of neural activity-dependent gene expression, we evaluated seasonal changes in the density of glutamatergic neurons in RA.

## Methods

### Animals

Male canaries of each season (first fall, second spring, and second fall) were acquired from the breeding colony at the Center for Field Research in Ethology and Ecology at Rockefeller University and from the colony at Hokkaido University. Canaries were kept in indoor breeding cages under a light/dark cycle mimicking local daylight conditions, which gradually changed 15 min per week.

### Song recording and analysis

Songs were recorded using a unidirectional microphone (SM57, Sure) and transferred to a computer using Sound Analysis Pro v1.04 (http://soundanalysispro.com/) [46]. During song recording sessions, each bird was individually housed in a cage inside a sound-attenuated box. The singing duration was defined as the total seconds of song bouts produced in 30 min before brain sampling. A song bout was defined as the continuous production of syllables, followed by at least 400 ms of silence.

The score of syllable similarity between adjacent syllables was calculated as the peak correlation coefficient between two syllables (Avisoft-SASLab Pro, Avisoft Bioacoustics). Song files used for this similarity analysis were recorded in 30-min singing sessions in the morning before brain sampling for that singing condition. For each bird, 7–10 song files were randomly selected from the recorded files to separate 300 syllables as individual “.son”-converted syllable files. A total of 300 serially separated syllable files were transferred to the Avisoft CORRELATOR program to calculate the similarity scores between the adjacent syllables. The two spectrograms of the separated syllables were shifted incrementally past each other along the time axis. For each offset position, the correlation coefficient was computed according to the following formula:

$$\Phi_{XY} = \frac{{\sum\limits_{X} {\sum\limits_{Y} {((a_{xy} - m_{a} )*(b_{xy} - m_{b} ))} } }}{{\sqrt {\sum\limits_{X} {\sum\limits_{Y} {(a_{xy} - m_{a} )^{2} *\sum\limits_{X} {\sum\limits_{Y} {(b_{xy} - m_{b} )^{2} } } } } } }}$$

where *m*_*a*_ and *m*_*b*_ are the mean values of the spectrograms *a* and *b*, respectively. *a*_*xy*_ and *b*_*xy*_ are the intensities of the spectrogram points at the locations *x* and *y*, respectively. The syllable similarity score is a value ranging from 0 to 1. A value of 1 means that the two spectrograms are identical, whereas a value of 0 means that there was no similarity between the spectrograms.

### Brain sampling

Male canaries of each season were grouped based on six experimental conditions: (I) first fall with 30 min of silence as silent control (n = 4 sampled on October 2 or 3 in 2006 and 2010), (II) first fall with 30 min of singing (n = 4 sampled from September 24 to October 2 in 2010), (III) second spring with 30 min of silence (n = 3 sampled from June 8 to 14 in 2009 and 2010), (IV) second spring with 30 min of singing (n = 6 sampled from June 2 to 15 in 2010 and 2015; for in situ hybridization with *Dusp6* and *Gadd45β*, n = 5 birds were used owing to the limitation of available brain sections), (V) second fall with 30 min of silence (n = 3 sampled from September 14 to October 4 in 2007, 2010, and 2015), and (VI) second fall with 30 min of singing (n = 6 sampled from September 17 to 27 in 2009 and 2015). Brain tissues were immediately sampled after a 30-min singing session in the morning because the time point with the highest expression of IEGs in song nuclei occurs before the start of mRNA degradation [36, 39, 43, 44]. After each singing behavior observation session, the birds were humanely killed by decapitation. The brains were embedded in OCT compound (Sakura Fine Technical) and stored at − 80 °C until use.

### In situ hybridization

In situ hybridization was performed as described previously [39]. cDNA fragments of *Arc*, *Egr1*, *c-fos*, *Nr4a1*, *Sik1*, *Dusp6*, and *Gadd45β* were cloned from a whole-brain cDNA mixture of a male zebra finch and used for the synthesis of in situ hybridization probes as described in a previous study [41]. Frozen sections that were 12-µm thick were cut in the sagittal plane. Brain sections for a given experiment were fixed with 3% paraformaldehyde in 1× phosphate-buffer saline (PBS), acetylated, dehydrated in ethanol solutions of increasing concentrations, and processed for in situ hybridization with antisense ^35^S-UTP labeled riboprobes of the target genes. Hybridization was performed at 65 °C overnight. After washes, the slides were dehydrated in ethanol solutions of increasing concentrations and exposed to an X-ray film (Biomax MR, Kodak) for 2–14 days. The slides were then dipped in an auto-radiographic emulsion (NTB2, Kodak), incubated for 2–8 weeks, and processed with a D-19 developer (Kodak) and fixer (Kodak). The developed slides were Nissl-stained with cresyl violet acetate solution (Sigma). To minimize experimental artifacts and variability in detection signals, we performed the fixation and acetylation of all sections tin the same batch containing brains of canaries from each season (first fall, second spring, and second fall), used the same solution of S^35^-radioisotope probes for each gene, and developed the signals on X-ray films and silver-dipped brain sections for the same amount of time [47]. The exposed X-ray films of the brain images were digitally scanned using a Z16 APO dissecting microscope (Leica) connected to a DFC490 CCD camera (Leica) with Application Suite V3 imaging software (Leica) at 50× magnification. The same light settings were used consistently across all images from each experiment. Photoshop (Adobe Systems) was used to measure mean pixel intensities in the brain areas of interest from at least two sections after their conversion to 256 grayscale images. The induction response of neural activity-dependent genes in each bird was calculated as follows:

$${\text{Induction response of neural activity-dependent gene}} = \frac{{\left( {{\text{Gene expression level of individual singing bird }}{-}{\text{ Average expression level of silent birds}}} \right)}}{{{\text{Total singing duration }}\left( {{\text{sec}}} \right){\text{ of the individual in }}30{\text{ min singing session }}}}$$

Fluorescent in situ hybridization was performed as previously described [41]; dinitrophenyl (DNP)- and digoxigenin (DIG)-labeled riboprobes were used for *Arc* and *Vglut2*, respectively. DNP-labeled probes were detected with an anti-DNP horseradish peroxidase (HRP)-conjugated antibody using a TSA DNP system (Perkin Elmer) and anti-DNP KLH AlexaFluor488 (A-11097, Molecular Probes). Following treatment with 1 × PBS containing 1% H_2_O_2_ for 30 min, DIG-labeled probes were detected with an anti-DIG HRP-conjugated antibody (200-032-156, Jackson Laboratory) and a TSA Plus Cy5 system (Perkin Elmer). The brain sections were then mounted in Vectashield with 4′,6-diamidino-2-phenylindole (DAPI) (Vector Laboratories Inc., Burlingame, CA, USA). Signal images were obtained by fluorescence microscopy using the EVOS FL Imaging System (Thermo Fisher Science).

### Statistical analysis

One-way ANOVA was used for analyzing the differences in singing amount and cell density among the three groups (first fall, second spring, and second fall). Differences in gene induction response for each gene among the different seasons (first fall, second spring, and second fall) and song nuclei were investigated using two-way ANOVA followed by Scheffe’s *F* tests when appropriate. Analysis of covariance (ANCOVA) was performed to evaluate the homoscedasticity from the regression line of the gene induction intensity between singing duration and expression level. Pearson’s correlation coefficient with Bonferroni correction was used for analyzing the relationship between the induction rate of gene expression and vocal plasticity. All raw data are available as Additional file 1.

## Results

### Seasonal difference in singing-driven *Arc* expression

We first examined the difference in the degree of vocal plasticity of canaries whose brain tissue was sampled after a 30-min singing session at the first fall, the second spring, and the second fall. The crystallized song of canaries has a phase structure formed by repetitive syllables. By contrast, such a phase structure with repetitive syllables was not observed in plastic songs with high vocal plasticity (Fig. 1c). Thus, we adopted the mean and coefficient of variation (CV) of similarity scores between adjacent syllables in songs as the behavioral parameters of vocal plasticity. We found a significant difference in both mean and CV of similarity scores between adjacent syllables in songs between groups from the spring and fall seasons (Fig. 1d, e). Although some individual differences in vocal plasticity were observed in the second fall canaries, both parameters of vocal plasticity were similar in the fall between the first year juvenile and the second year adult canaries. In addition, we confirmed that although there was a trend that the total singing duration was longer in birds singing in the spring than in the fall, there were no significant differences in the total singing amount in the 30-min sessions among the three groups (one-way ANOVA, ^n.s.^*p* > 0.05) (Fig. 1f).

**Fig. 2:**
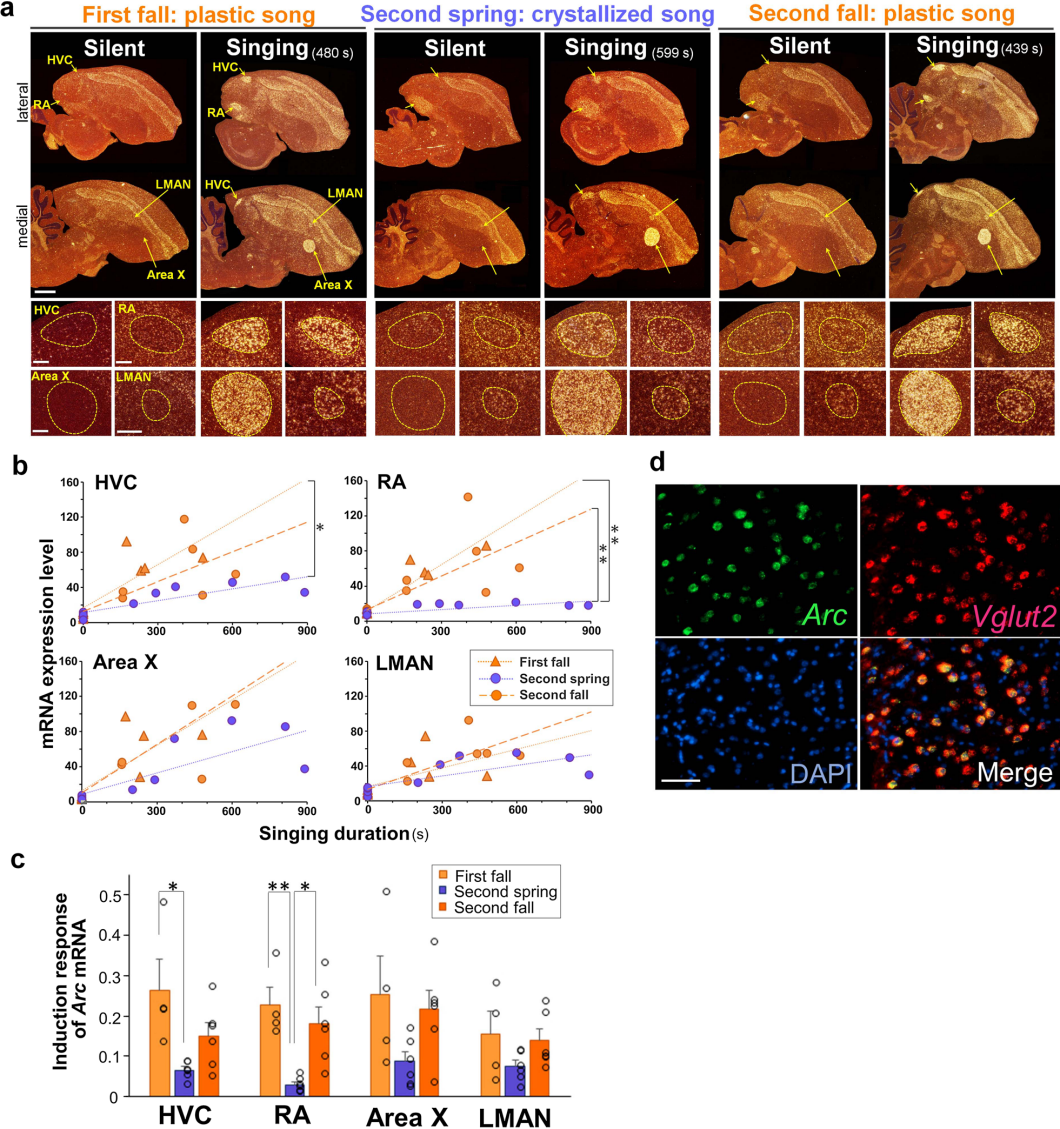
Seasonal changes in singing-driven *Arc* expression in the song nuclei of canaries. **a** Singing-driven *Arc* mRNA expression in first fall, second spring, and second fall groups. (Upper panels) Total singing duration (s) before brain sampling is shown on the panels of each bird. White dots: *Arc* mRNA expression. Red: cresyl violet counterstain. Scale bar = 1.5 mm. (Lower panels) Higher-magnification images of *Arc* mRNA expression in HVC, RA, Area X, and LMAN from the same birds represented in the upper panels. Scale bar = 200 μm. **b** *Arc* mRNA expression in HVC, RA, Area X, and LMAN in the 30-min singing session. Orange circles: first fall (silent n = 4 and singing n = 4 birds); blue circles: second spring (silent n = 3 and singing n = 6 birds); and orange triangles: second fall (silent n = 3 and singing n = 6 birds). ANCOVA. *p < 0.05, **p < 0.01. **c** Induction response of *Arc* mRNA expression in song nuclei after singing in first fall (light orange, n = 4), second spring (blue, n = 6), and second fall (dark orange, n = 6) singers. Two-way ANOVA with Scheffe’s F test. *p < 0.05, **p < 0.01. **d** Selective expression of singing-driven *Arc* mRNA in excitatory glutamatergic neurons with *Vglut2* in RA (green and red, respectively). DAPl-stained nuclei are shown in blue. Scale bar = 40 μm

We then investigated the singing-driven expression of the neural activity-dependent cytoskeleton-associated protein *Arc* (also called *Arg3.1*) among the first fall, second spring, and second fall groups. *Arc* is a neural activity-dependent neuroplasticity-related IEG that is induced by singing-driven neural activity in song nuclei with the highest sensitivity and intensity to other singing-driven IEGs [38, 39, 41, 44]. Singing-driven *Arc* mRNA expression was examined in four major telencephalic song nuclei—HVC, RA, LMAN, and Area X (Fig. 2a). The thalamic song nucleus DLM was excluded in this analysis because of the nondetectable induction of most IEGs, including *Arc*, by singing in this nucleus [39, 44]. During all three periods, *Arc* mRNA expression was induced after 30 min of singing in all four song nuclei compared with the silent condition (Fig. 2b). However, the expression patterns of *Arc* between the song nuclei were inconsistent throughout the three seasons. To evaluate the quantitative expression level of singing-driven *Arc* at the two seasonal time points and different ages, we examined the relationship between the *Arc* expression in each song nuclei and the total singing duration of individual birds (Fig. 2b). In HVC and RA, we found that the level of singing-driven *Arc* expression was significantly different between fall and spring (first fall vs. second spring in HVC, *F* (1, 17) = 6.54, *p* = 0.02; first fall vs. second spring in RA, *F* (1, 15) = 11.49, *p* = 3.4e−3; and second spring vs. second fall in RA, *F* (1, 18) = 11.49, *p* = 3.4e−3). By contrast, song nuclei in the AFP, Area X, and LMAN did not show statistically different expression of *Arc* mRNA among the three groups, albeit the presence of trends showing higher expression of *Arc* in the first and second fall than in the second spring. These results were confirmed as a comparison of the induction responses of *Arc* expression, which were calculated as the increased expression of *Arc* mRNA per total singing duration (s) in a 30-min singing session for individual singers (see Methods). The induction response of *Arc* mRNA was significantly different in HVC and RA between the first fall and second spring groups. Furthermore, the degree of *Arc* induction response was different only in RA between second spring and second fall groups (two-way ANOVA with Scheffe’s F test. *p < 0.05, **p < 0.01) (Fig. 2c). Especially in RA, we found that the induction response of *Arc* was higher in singing canaries with plastic songs in both the first and second fall than in singing canaries with crystallized songs in the second spring. Additionally, as shown in the zebra finch [41], *Arc* expression was induced by singing selectively in glutamatergic excitatory neurons stained with a *Vglut2* probe in RA (mean ± SD: 97.4 ± 2.4%, n = 4 birds) (Fig. 2d), which are considered the projection neurons from RA to the downstream nXIIts. These results indicated seasonal differences in *Arc* induction response in the vocal motor song nuclei HVC and RA.

### Different expression of other neural activity-dependent genes by seasonal singing

**Fig. 3:**
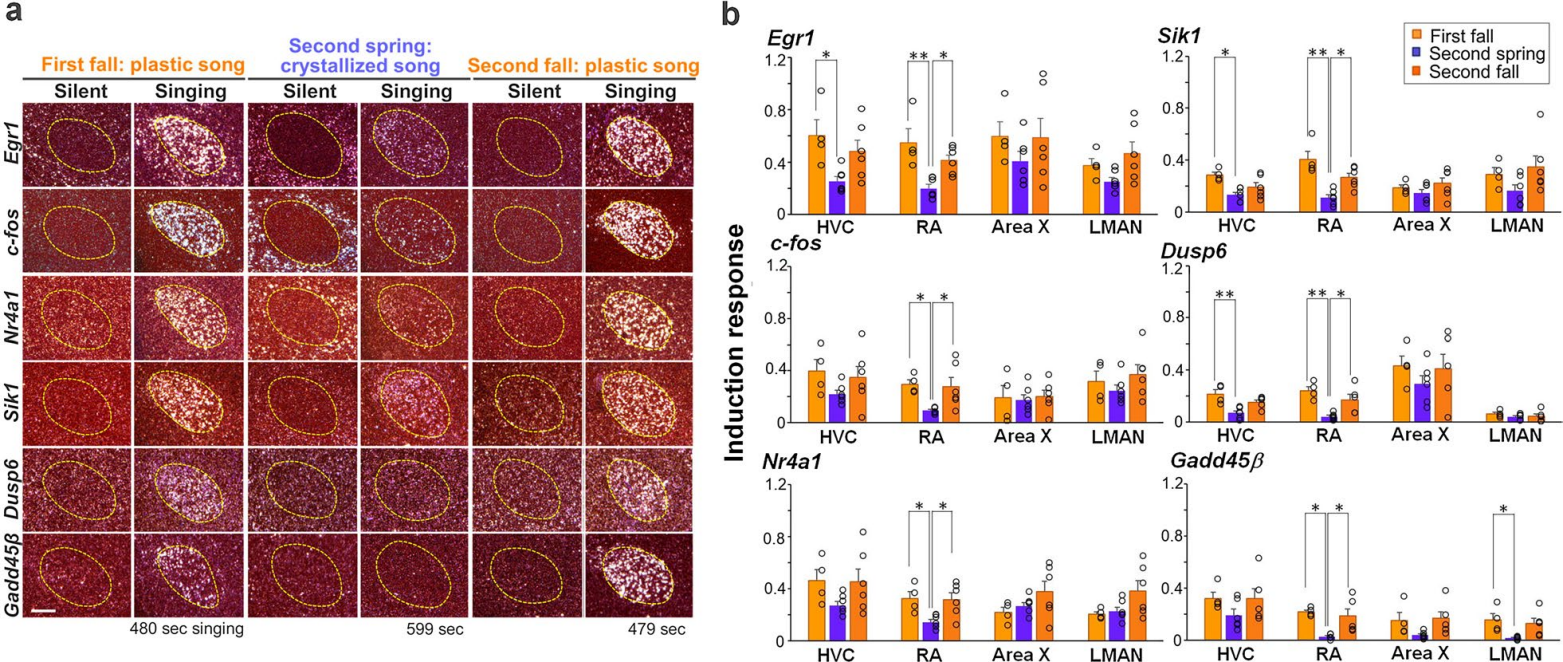
Differential expression of singing-driven IEGs during the fall and spring. **a** Examples of singing-driven induction of IEG mRNAs (*Egr1*, *c-fos*, *Nr4a1*, *Sik1*, *Dusp6*, and *Gadd45β*) in RA in first fall, second spring, and second fall. All images in columns are from the same bird. Total cumulative singing duration before brain sampling is shown at the bottom of the panels. White dots: mRNA expression. Red: cresyl violet counterstain. Scale bar = 200 μm. **b** Induction response of the six singing-driven IEGs in song nuclei in first fall, second spring, and second fall. Two-way ANOVA with Scheffe’s F test. *p < 0.05, **p < 0.01

Our findings on *Arc* led us to consider other neural activity-dependent genes, which we previously identified as singing-driven genes associated with the vocal plasticity state in the zebra finch, a closed-ended learner [41]. In neural activity-dependent genes, there are two major groups: transcription factors for inducing downstream genes and direct functional effectors for cellular signaling and plasticity [35, 48, 49]. We chose six genes associated with vocal plasticity in the zebra finch: *Egr1* (early growth response 1) [50, 51], *c-fos* [52, 53], and *Nr4a1* (nuclear receptor subfamily 4 group A member 1) [54, 55] as transcription factor IEGs and *Sik1* (salt inducible kinase 1) [56, 57], *Dusp6* (dual-specificity phosphatase 6) [58, 59], and *Gadd45β* (growth arrest and DNA-damage-inducible protein beta) [60, 61] as functional effector IEGs. Similar to *Arc* expression, these genes showed different expression patterns and levels after singing among the song nuclei through the two seasonal time points and different ages. Especially in RA, we found that the expression level of singing-driven genes was different between spring and fall (Fig. 3a). In a manner consistent with differences in absolute expression level, we also found that the induction responses of all tested genes were significantly higher in RA in the first and second fall than in the second spring (two-way ANOVA with Scheffe’s F test. *p < 0.05, **p < 0.01) (Fig. 3b). However, in other song nuclei, the induction responses of most of these other genes did not show consistent differences between seasons, although there was a similar trend toward being higher in the fall than in the spring. These results showed that not only *Arc* but also other neural activity-dependent genes were more intensely induced in the song nucleus RA by singing in the fall than in the spring. Furthermore, in the fall, the level of induction responses of singing-driven IEGs was similar between the first and second years.

### Induction response of singing-driven IEGs in RA associated with song vocal plasticity

**Fig. 4:**
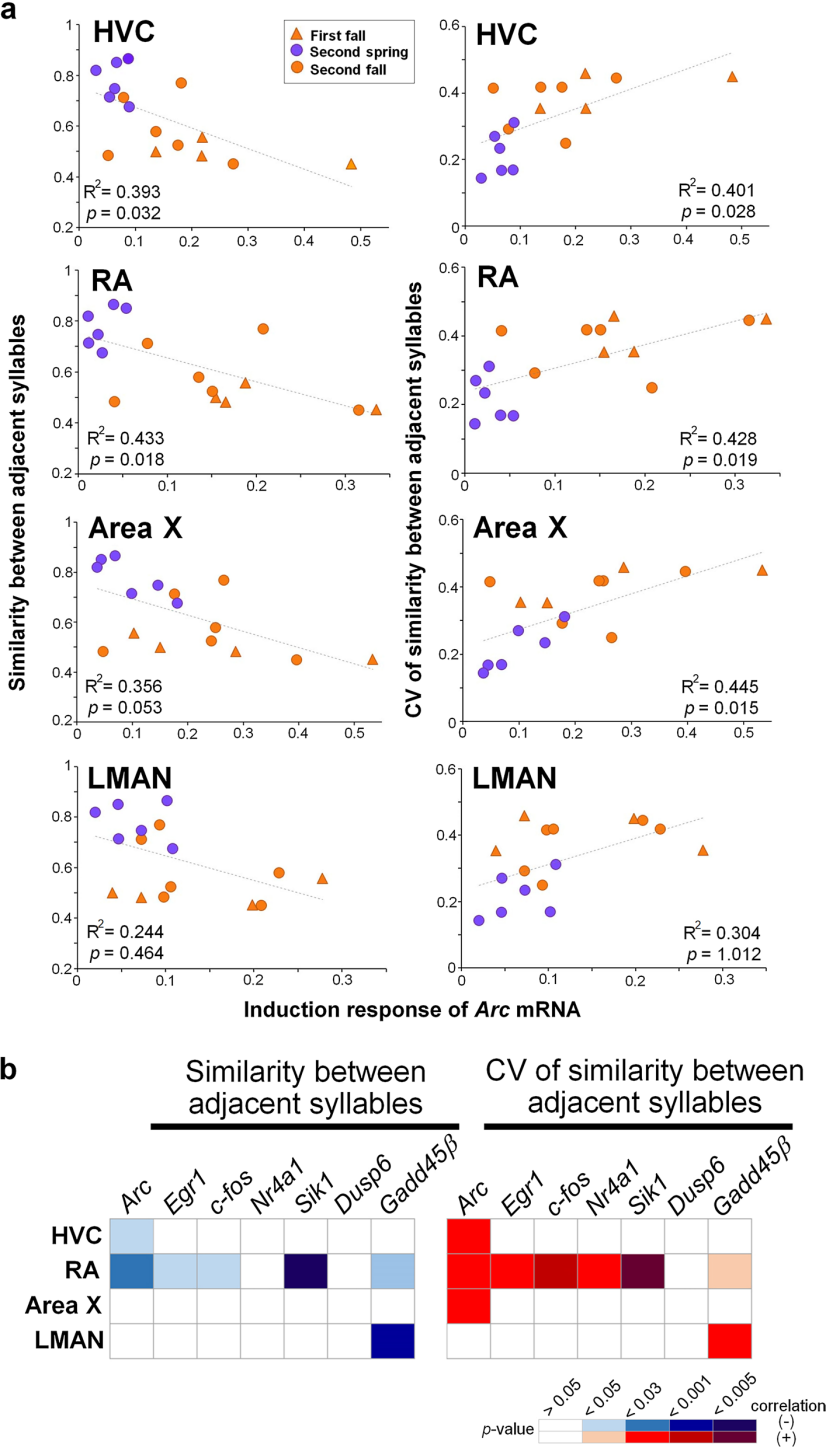
Correlation between singing-driven induction of neural activity-dependent genes and song plasticity. **a** Correlation between the induction response of singing-driven *Arc* mRNA expression in song nuclei and vocal plasticity. Left and right columns: mean and CV of similarity between adjacent syllables (300 syllable sets), respectively. Orange triangles, purple circles, and orange circles represent birds with singing in first fall (n = 4), second spring (n = 6), and second fall (n = 6), respectively. **b** Heat maps showing correlation values in song nuclei between singing-driven expression of the seven activity-dependent IEGs and song plasticity. Left and right panels: mean and CV of similarity between adjacent syllables, respectively. Pearson’s correlation coefficient with Bonferroni correction

We then examined a possibility that the induction responses of singing-driven IEGs were not just different between seasons but also associated with the state of vocal plasticity, which was seasonally regulated. To investigate this, we performed a correlation analysis between the induction responses of singing-driven IEGs and the states of vocal plasticity across individual singers at the two seasonal time points and different ages. Consequently, we found that the induction responses of singing-driven *Arc* expression in HVC, RA, and Area X showed significant correlations with the behavioral phenotypes of vocal plasticity (Pearson’s correlation coefficient with Bonferroni correction) (Fig. 4a). The induction response of *Arc* was negatively correlated with mean similarity scores between adjacent syllables (Fig. 4a, left panels). By contrast, it was positively correlated with the CV of the similarity scores (Fig. 4a, right panels). Similarly, except for *Dusp6*, the induction responses of all other singing-driven IEGs in RA showed consistently significant associations with either mean or CV of the similarity scores between adjacent syllables through the seasons (Fig. 4b). These results indicated that seasonal differences in the induction of singing-driven IEGs in RA reflect some degree of vocal plasticity during singing.

### Seasonal consistency in the number of glutamatergic excitatory neurons in RA

**Fig. 5:**
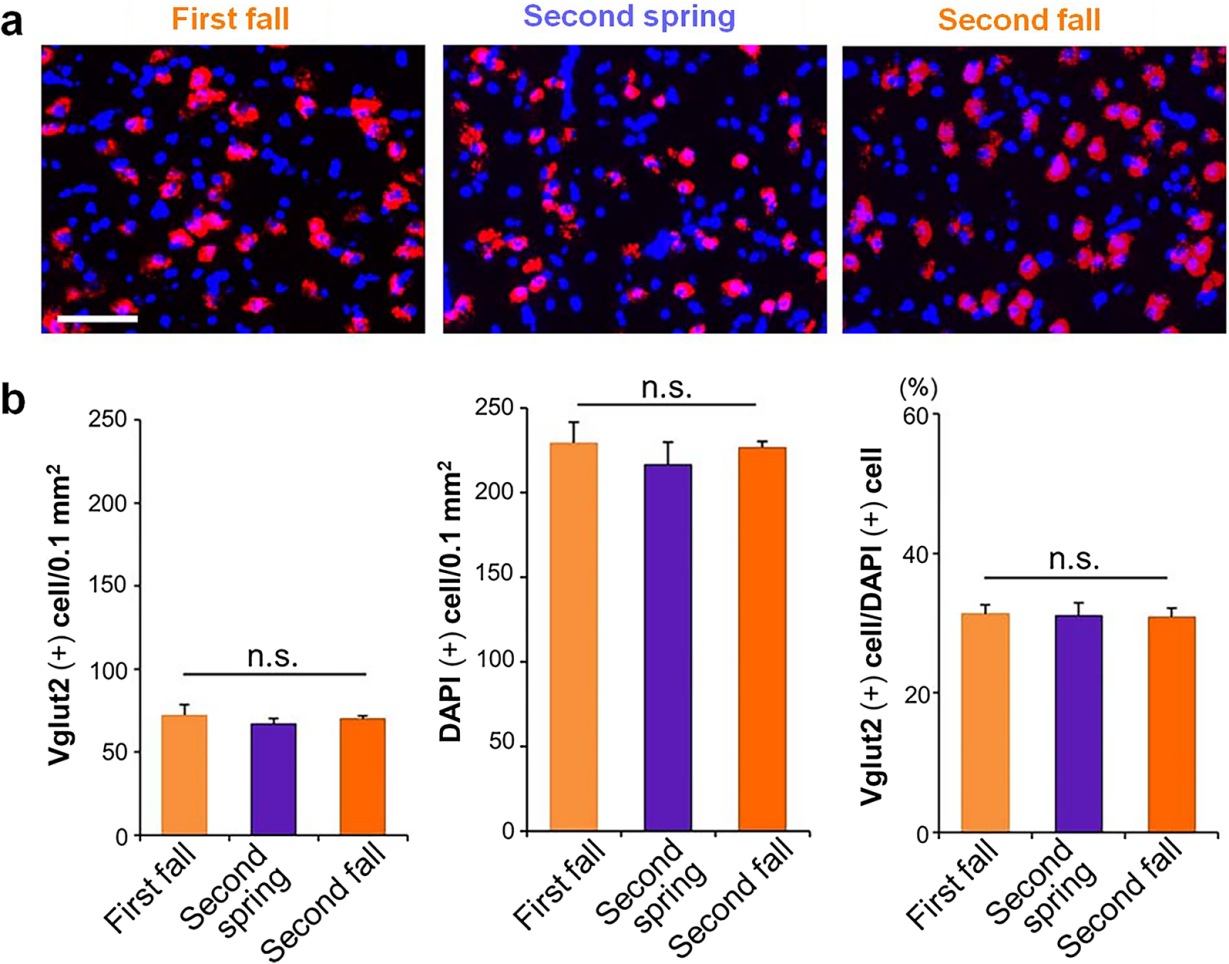
Consistent number and component of glutamatergic neurons in RA. **a** Typical images of a cell component in RA in first fall, second spring, and second fall. Blue: cell nuclei stained with DAPI, Red: *Vglut2* (+) cells. Scale bar = 50 μm. **b** No significant differences between *Vglut2* (+) cell number, whole cell number, and *Vglut2* (+) cell density in RA were found between first fall, second spring, and second fall birds (each season = 3 birds). One-way ANOVA. ^n.s.^*p* > 0.05

To evaluate whether differences in the expression of singing-driven IEGs in RA are caused by seasonal differences in cell number or density between the first fall, second spring, and second fall, we investigated these values of RA glutamatergic neurons. Because of the selective expression of singing-driven *Arc* in glutamatergic neurons in RA (Fig. 2d), we counted *Vglut2* (+) cells and DAPI-stained cell nuclei to assess the number of glutamatergic neurons and total cells in RA. We confirmed that there were no significant differences in glutamatergic neuron density, total cell density, and population ratio in RA across the three seasons (one-way ANOVA, ^n.s.^*p* > 0.05) (Fig. 5). These results indicated that seasonal differences in the expression of singing-driven genes were not affected by seasonal differences in neuronal cell number or density in RA.

## Discussion

The developmental production of vocal plasticity, such as in plastic song singing, is regulated species-specifically, particularly between closed- and open-ended song learners in songbirds [62, 63], and is an essential behavioral state proceeding the sensorimotor learning of their songs [64, 65]. In this study, we revealed that canaries, open-ended song learners, show seasonal differences in the induction response of neural activity-dependent IEG expression by singing. In contrast to neurogenesis, which is seasonally regulated in HVC of canaries [27, 28], the seasonal difference in singing-driven expression of IEGs was consistently observed in the vocal nucleus RA, with higher induction by singing in the fall than in the spring. The induction response of singing-driven IEGs in RA is linked to the degree of vocal plasticity through the seasons.

In previous studies on zebra finches, a closed-ended learner, vocal learning plasticity in juveniles was accompanied by the intense induction of the singing-driven IEGs, including *Arc*, in the glutamatergic projection neurons of RA with dense dendritic spines [41, 44]. Synaptic plasticity in RA-projecting neurons is modulated by activity-dependent long-term depression (LTD), which is selectively induced during the critical period of vocal sensorimotor learning in juvenile zebra finches [66]. Similarly, in mammals, *Arc* is an activity-dependent modulator of synaptic plasticity during LTD [67, 68]. Although canaries from the first and second fall sang plastic songs (i.e., at a high vocal plasticity state), not only *Arc* but also other effector IEGs such as *Sik1*, *Dusp6*, and *Gadd45β* were intensively expressed in RA (Figs. 2 and 3). In zebra finches, these IEGs are selectively expressed in the glutamatergic RA-projecting neurons [41]. Similarly, in canaries, *Arc* was expressed selectively in the glutamatergic neurons of RA (Fig. 2d). However, the expression of other IEGs in the glutamatergic neurons of RA was not examined. These findings suggest their potential molecular function in neuronal signaling and plasticity of RA-projecting neurons, which may directly regulate seasonal vocal plasticity.

In the canary, because of seasonally regulated adult neurogenesis and cell death in HVC, certain populations of older HVC_(RA)_ neurons are replaced by newly incorporated HVC_(RA)_ neurons in the fall [28, 69]. The turnover of HVC_(RA)_ neurons induces the expansion of new axons from the incorporated HVC_(RA)_ neurons into RA and the pruning of older HVC–RA synaptic connections. Hence, a large number of synaptic connections between the HVC_(RA)_ neurons and RA-projecting neurons are rearranged in the fall. In this study, we found that Area X and LMAN in canaries have relatively consistent induction of singing-driven IEG expression throughout the three seasons, similar to zebra finches during the critical period of song learning [41], suggesting a consistent degree of neural firing during singing in the AFP nuclei over the course of a year. The AFP-driven input signal from LMAN to RA has crucial roles in generating exploratory variability [15, 70, 71]. Thus, the AFP-driven input signal for vocal fluctuation could be consistently conveyed via the LMAN–RA synaptic connection throughout the seasons. If so, synaptic balances between HVC–RA and LMAN–RA connections may be variable owing to seasonal HVC_(RA)_ neuron turnover. The LMAN–RA synaptic connections could be stronger and more in number than the HVC–RA connections in every fall, which is a state similar to that in juvenile zebra finches [65, 70, 72]. Therefore, the intense expression of singing-driven IEGs in RA glutamatergic neurons in the fall could play an important role in regulating the activity-dependent synaptic plasticity underlying the reconstruction of HVC–RA connections, which consequently leads to song modification in adulthood. The possibility of a functional link between neural activity-dependent gene expression and neurogenesis has not been directly examined yet, and RA-projection neuron-specific gene knockout/knockdown experiments for these genes will be essential in investigating this phenomenon in detail in the future.

To facilitate the understanding of neural molecular mechanisms underlying species-specific learned behavior from an evolutionary perspective, it could be useful to consider a concept from evolutionary developmental biology—species-specific spatiotemporal expression of limited numbers of transcriptional regulators generates diverse morphological phenotypes as “developmental toolkits” by switching the expression of other regulatory and structural genes on/off [73, 74]. Similarly, various species-specific phenotypes of learned behaviors are generated by temporally different expressions of regulatory genes for neural excitation/inhibition and plasticity in evolutionally conserved neural circuits [75]. To support this idea, a similar set of singing-driven IEGs is induced in song nuclei during high vocal plasticity states in both the canary and zebra finch [41, 44, 45]. Whereas the zebra finch possesses the regulatory switch for increasing the induction of singing-driven IEGs in RA at a single developmental period after hatching, the canary possesses the trait to reinduce these genes at multiple seasonal points throughout its life. With regards to the transcriptional regulatory mechanisms underlying the species-specific expression of singing-driven IEGs, the dynamics of sex hormones, especially testosterone concentration, could play an important role in the modification of neuronal activity and activity-dependent gene expression in RA. Although no studies have examined neural activity during singing through different seasons and ages in the canary, it has been previously reported that, in the zebra finch RA, NMDA receptor-mediated excitatory postsynaptic currents (NMDA-EPSCs) become faster throughout the critical period of song development and testosterone treatment of juveniles induces NMDA-EPSCs to become prematurely fast in RA [76]. Ca^2+^ influx through NMDA receptors triggers the induction of IEG transcription via the Ca^2+^ signaling pathway [77, 78]. Indeed, juvenile zebra finches treated with testosterone have a decreased induction response of singing-driven *Arc* expression in RA and, accordingly, hasten song stabilization at an early developmental phase compared with normal birds [44]. For many open-ended learners, including the canary, testosterone concentration is low during the fall and winter and becomes higher in the following spring and summer [79–81]. Hence, the induction responses of singing-driven IEGs in RA could be regulated via an inverse relationship with the seasonal concentrations of testosterone in the canary, suggesting that species differences in testosterone concentration during development regulate the species-specific induction responses of singing-driven IEGs in song nuclei. Thus, the expression differences in singing-driven transcription factors/modulators, such as *Egr1*, *c-fos*, and *Nr4a1*, could play a role as “neural toolkit” genes for organizing species-specific vocal learning programs by regulating their downstream effects on neural plasticity. As a candidate for such downstream molecules of singing-driven IEGs, the maturation of extracellular matrix perineuronal nets (PNNs) in song nuclei is associated with song vocal plasticity in both open- and closed-ended songbirds [79, 82, 83]. PNNs are important molecules contributing to the closure of developmentally critical periods of the visual system by regulating parvalbumin-expressing interneuron signaling [84–87].

From an evolutionary perspective, further studies on gene expression in the song circuits of other songbird species that possess species-specific phases and programs for song learning would meaningfully add to our understanding of the regulation involved during the critical period of vocal learning. Additionally, comparative genomics of the transcriptional regulatory regions in such species-specifically regulated genes expressed in song nuclei would be an important research direction for studying the evolution of learned behavior.

## Conclusions

We revealed that canaries, open-ended song learners, show seasonal differences in singing-driven induction response of neural activity-dependent IEGs (*Arc*, *Egr1*, *c-fos*, *Nr4a1*, *Sik1*, *Dusp6*, and *Gadd45β*). The seasonal difference in singing-driven expression of IEGs was observed in the vocal nucleus RA, with higher induction by singing in the fall than in the spring, even at different ages. In addition, the induction response of singing-driven IEGs in RA is linked to the degree of song plasticity through the seasons. These results indicate a potential relationship between the singing-driven IEG expression level and the degree of vocal plasticity in an age-independent manner.

## Supplementary Information

**Additional file 1:** The quantified numerical data for each sample used for statistical analyses.

## Data Availability

The authors confirm that all data underlying the reported findings are included in the manuscript. All raw data are available as Additional file 1.
